# Haemolysis and haem oxygenase-1 induction during persistent “asymptomatic” malaria infection in Burkinabé children

**DOI:** 10.1186/s12936-018-2402-6

**Published:** 2018-07-06

**Authors:** Jason P. Mooney, Aissata Barry, Bronner P. Gonçalves, Alfred B. Tiono, Shehu S. Awandu, Lynn Grignard, Chris J. Drakeley, Christian Bottomley, Teun Bousema, Eleanor M. Riley

**Affiliations:** 1 Department of Immunology and Infection, London School of Hygiene and Tropical Medicine, London, UK; 2 The Roslin Institute and Royal (Dick) School of Veterinary Studies, University of Edinburgh, Easter Bush, Midlothian, EH25 9RG UK; 3 Centre National de Recherche et de Formation sur le Paludisme, Université de Ouagadougou, Ouagadougou, Burkina Faso; 4 Department of Infectious Disease Epidemiology, London School of Hygiene and Tropical Medicine, London, UK; 5 Radboud Institute for Health Sciences, Radboud University Medical Center, Nijmegen, The Netherlands

**Keywords:** Subclinical malaria, Anaemia, Haemolysis, IL-10, HO-1, Burkina Faso

## Abstract

**Background:**

The haemolysis associated with clinical episodes of malaria results in the liberation of haem, which activates the enzyme haem oxygenase-1 (HO-1). HO-1 has been shown to reduce neutrophil function and increase susceptibility to invasive bacterial disease. However, the majority of community-associated malaria infections are subclinical, often termed “asymptomatic” and the consequences of low-grade haemolysis during subclinical malaria infection are unknown.

**Study design and results:**

As part of an ongoing study of subclinical malaria in Burkina Faso, 23 children with subclinical *Plasmodium falciparum* infections (determined by qPCR) were compared with 21 village-matched uninfected control children. Infected children showed evidence of persistent haemolysis over 35 days, with raised plasma haem and HO-1 concentrations. Concentrations of IL-10, which can also directly activate HO-1, were also higher in infected children compared to uninfected children. Regression analysis revealed that HO-1 was associated with haemolysis, but not with parasite density, anaemia or IL-10 concentration.

**Conclusions:**

This study reveals that subclinical *P. falciparum* malaria infection is associated with sustained haemolysis and the induction of HO-1. Given the association between HO-1, neutrophil dysfunction and increased risk of Salmonella bacteraemia, prolonged HO-1 induction may explain epidemiological associations and geographic overlap between malaria and invasive bacterial disease. Further studies are needed to understand the consequences of persistent subclinical malaria infection, low-grade haemolysis and raised HO-1 on immune cell function and risk of comorbidities.

## Background

In malaria endemic areas, the vast majority of malaria infections that are detected in community surveys are ‘asymptomatic’, that is they do not present with fever or other obvious clinical signs that prompt treatment-seeking behaviour. Individuals carrying these infections are often referred to as being ‘clinically immune’. However, *Plasmodium falciparum* parasites, sometimes present at very low densities, can be detected in the peripheral blood of these individuals either microscopically or by highly sensitive PCR [1–3]. Asymptomatic infections may perpetuate malaria transmissions and, as individuals rarely seek treatment, can act as a reservoir of ‘cryptic’ transmission, undermining efforts at malaria elimination [4, 5]. Moreover, there is accumulating evidence that these infections may be harmful to the infected individual; being associated with, for example, reduced school attendance and cognitive ability [6], strengthening the argument that they should be referred to as subclinical rather than asymptomatic and should be treated [3].

Acquired immunity to malaria develops after repeated exposure but is non-sterilizing; clinically immune individuals harbour frequent, persistent or recurrent infections throughout their lives [7–11]. These subclinical infections have been associated with persistent low-grade haemolysis, and fluctuations in parasite density may result in intermittent, higher density parasitaemia and further haemolysis [12, 13]. Over time, extensive destruction of both parasitized and non-parasitized red blood cells [14, 15] can lead to moderate and even severe anaemia [16]. Very low parasite densities can also lead to diagnostic confusion given the numerous comorbidities that may occur in malaria endemic regions [17].

Associations between bacteraemia (especially due to non-Typhoidal *Salmonella*, NTS), malaria infection and anaemia have been noted since 1929 [18, 19], with the first large case series published from The Gambia in 1987: seventy percent of NTS bacteraemia patients were anaemic and carried malaria parasites, typically at very low densities [20]. Recently, the molecular and cellular basis for malaria and NTS coinfection has been elucidated. Experiments in mice have shown that the immunoregulatory cytokine interleukin (IL)-10 impairs control of *Salmonella* through reduced neutrophil migration into infected tissues [21–23]. Further, malaria-induced haemolysis results in liberation of haem, a highly toxic pro-oxidant that is degraded by haem oxygenase-1 (HO-1). In turn, HO-1 impairs the function of circulating neutrophils in mice [24]; this impaired neutrophil function leads to increased susceptibility to invasive bacterial disease [21, 24]. Importantly, raised IL-10, HO-1 and impaired neutrophil respiratory burst have also been observed in children recovering from an acute symptomatic malaria infection [25]. However, it is not clear to what extent these defects are also induced by low density, subclinical malaria infections and/or may underlie the high incidence of invasive bacterial infections observed in malaria endemic areas.

In this preliminary study, the hypothesis was that subclinical malaria infection causes persistent haemolysis and low grade inflammation leading to raised HO-1 and inflammatory and/or regulatory cytokines. Plasma concentrations of markers of haemolysis and inflammation were compared among 23 children from Burkina Faso with subclinical *P. falciparum* infections and 21 village-matched, uninfected, control children. Subclinical *P. falciparum* infection was associated with persistent haemolysis with elevated concentrations of IL-10 and HO-1. These findings suggest that further exploration of haemolysis, immune function and susceptibility to bacterial coinfection in ‘asymptomatically’ infected children is warranted.

## Methods

### Study population

The study was conducted in Balonghin in southwest Burkina Faso, a region of intense and highly seasonal malaria transmission occurring between June and October each year [26]. Two groups of children aged 5–10 years were surveyed. The first group was surveyed at the end of the dry season (June–July 2015); these children were included in the current analyses if they were free of infection by microscopy (reading 100 microscopic fields) and by 18 s qPCR [27]. The second group was surveyed on a monthly basis during the peak malaria season (September–December 2016). Children from this second cohort were included in the current analyses if they had chronic subclinical malaria infections (defined as two consecutive positive qPCRs 1 month apart in the absence of measured or reported fever) and samples were taken on the day this condition was met (d0, moment at which PCR + infections were present for at least 1 month) and 35 days later. After this last sample was collected, children received a full curative course of artemether–lumefantrine to clear their infections.

### Ethics and sample collection

Informed consent was provided by the parent or guardian of each child. The study was approved by the ethics committees of the London School of Hygiene and Tropical Medicine (reference number 9008) and the Ministry of Health in Burkina Faso (reference number 2015-3-033). Venous blood was collected into RNAprotect, heparin and EDTA-containing vacutainers. Whole blood samples were collected for parasitology from the EDTA tube. The blood from the heparin tube was centrifuged, and the plasma layer aspirated, aliquoted and stored at − 80 °C until use. Unfortunately, no leucocytes were collected for functional analysis.

### *Plasmodium falciparum* PCR

A MagNAPure LC automatic extractor (Roche Applied Science) was used to extract total nucleic acids from 100 µL venous blood samples collected in RNAprotect. Parasite density was quantified using 18 s qPCR [28] and serially diluted ring-stage NF54 parasites as the positive control [29].

### Haemoglobin

Haemoglobin concentrations were determined by self-calibrating Hemocue photometer (HemoCue 301 + , Angelholm, Sweden). Anaemia was defined using WHO guidelines for circulating levels of haemoglobin (g/dL) [30]. For children 5–11 years of age these are: non-anaemic (> 11.5), mild (11–11.4), moderate (8–10.9), and severe (< 8) anaemia.

Haemoglobin genotypes were determined in 21 of the 23 infected patients. Extracted DNA was amplified using previously published primers (300 nM each) [32]. After amplification, primers and nucleotides from the genomic PCR were removed using ExoSAP-IT reagent (Applied Biosystems). Cleaned PCR product was added to an ASPE (allele specific primer extension) reaction mix containing 250 nM of each ASPE probe and 200 µM Biotin-14-dCTP (Invitrogen). ASPE products were hybridised to 2500 beads (Luminex corp., The Netherlands) of each set, washed twice in 1 × Tm buffer (0.2 M NaCl, 0.1 M Tris, 0.8% TritonX-100, pH 8.0) and incubated for 15 min at 37 °C in 1 × Tm containing 0.1% bovine serum albumin (BSA) and 2.5 µg/mL Streptavidin, R-Phycoerythrin Conjugate (SAPE, Invitrogen). Fifty µl of the hybridized beads were read on a Magpix (Luminex corp., The Netherlands) and genotypes were called according to fluorescent signal. Probe sequences were as follows;

HbA(C) (5′CTTAAACTCTACTTACTTCTAATTCATGGTGCATCTGACTCCTG3′, bead set MTAG-A056),

HbC (5′AACTTTCTCTCTCTATTCTTATTTCATGGTGCATCTGACTCCTA3′, bead set MTAG-A043),

HbA(S) (5′CTAAACATACAAATACACATTTCACAGTAACGGCAGACTTCTCCT3′, bead set MTAG-A062),

HbS (5′AATCAACACACAATAACATTCATACAGTAACGGCAGACTTCTCCA3′, bead set MTAG-A048). This methodology is described in detail elsewhere (Grignard et al. pers.comm.).

### Plasma protein quantification

Enzyme-linked immunosorbent assays (ELISA) were conducted according to the manufacturers’ instructions to measure plasma concentrations of haptoglobin (HPT, GWB-8DA44B, Genway Biotech), haemopexin (HPX, GWB-4B6D1A, Genway Biotech), haem oxygenase-1 (HO-1, ADI-EKS-800, Enzo Life Sciences), transferrin (Tf, ab108911, abcam), and ferritin (ab108837, abcam). Colorimetric determination of haem in plasma samples was conducted according to the manufacturer’s instructions (DIHM-250, Bioassay Systems). Samples were diluted prior to testing as follows: 1:50,000 (HPT); 1:1 (haem), 1:40,000 (HPX), 1:10 (Ferritin), 1:6.6 (HO-1), and 1:200 (Tf). Plasma cytokine concentrations were determined by magnetic bead multiplex assay (EPX01A-10228-901 (IFN-γ), EPX01A-10215-901 (IL-10), EPX01A-12001-901 (G-CSF), EPX01A-10213-901 (IL-6), EPX01A-10223-901 (TNFα), EPX01A-10224-901 (IL-1β), EPX01A-10225-901 (IL-4), EPX01A-10204-901 (IL-8), ProcartaPlex Multiplex Immunoassay, Invitrogen, USA) following the manufacturer’s instructions, and analysed on a Luminex 100 (LuminexCorp, Austin, USA) running Bioplex Manager software. Samples were diluted 1:2.5. Samples giving values below the limit of detection were arbitrarily assigned a concentration at the limit of detection for the purposes of statistical analyses (13.008 pg/mL for IFN-γ, 9.5146 pg/mL for IL-10).

### Statistical analysis

Concentrations of cytokines and markers of haemolysis (haem, HPX, HO-1) were compared between uninfected controls and infected (PCR-positive) children at enrollment using the Mann–Whitney test. Correlations and partial correlations were calculated using the combined log-transformed data (day 1 and day 35). For the latter analysis, p-values were calculated using robust standard errors to account for repeated measures and within-individual correlation. Analyses were conducted using GraphPad Prism 7 and Stata version 14.

## Results

### Children with subclinical *Plasmodium falciparum* infections have persistent haemolysis

**Fig. 1:**
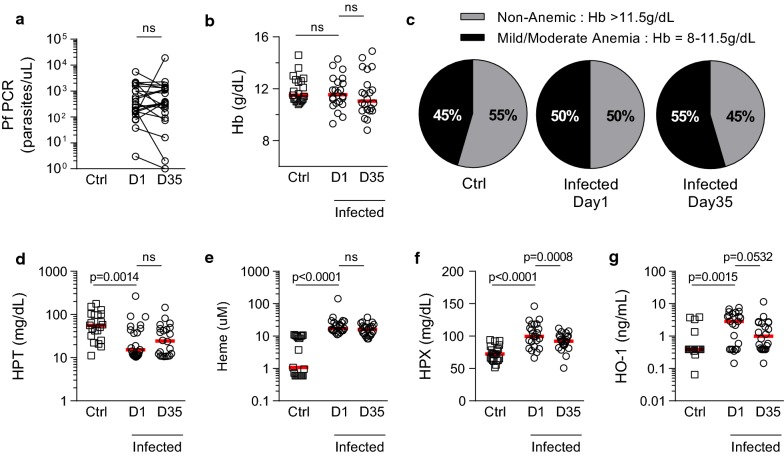
Haemolysis in children with subclinical malaria. At study enrollment (Day 1) or follow-up (Day 35), **(a)** *Plasmodium falciparum* (Pf) burden was determined by 18S qPCR for uninfected controls (Ctrl) (n = 21) or subclinically malaria-infected (n = 23) children (Table 1). **b** Haemoglobin (Hb) levels were determined by Hemocue analyzer. **c** Pie charts show proportion (%) of children with differing levels of anaemia (WHO classification: non-anaemic (> 11.5 g/dL), mild (11–11.5 g/dL), and moderate (8–10.9 g/dL)]. Plasma concentration of **(d)** haptoglobin (HPT), **(e)** haem, **(f)** haemopexin (HPX), or **(g)** haem oxygenase-1 (HO-1) determined by ELISA. Dot plots show individual patient parameters. Red lines represent medians. P-values for comparisons between uninfected controls (Ctrl) and infected children at enrollment were determined by Mann–Whitney test. P-values for comparisons between infected children at days 1 and 35 were determined by Wilcoxon matched-pair test. ‘ns’ indicates P > 0.05

At enrollment, the parasite densities of the 23 *P. falciparum* qPCR positive children ranged from 1 to 18967 parasites/µL (Table 1). Twenty-two of the children (95.6%) were still qPCR positive 35 days later and there was no consistent or statistically significant change in parasite density over that 5 week period (Fig. 1a, Table 1). Twenty-one children who were negative by microscopy and qPCR at enrollment were designated uninfected. Mean haemoglobin concentrations did not differ between uninfected and infected children either at enrollment or at follow up, although there was a trend for mean Hb to decline over the follow up period in infected children (Fig. 1b). None of the children were severely anaemic (defined as Hb < 8 g/dL) and the proportion of children with mild or moderate anaemia did not differ significantly between infected (50%) and uninfected (45%) children (Fig. 1c). Nevertheless, there was clear evidence of haemolysis among the infected children but not among the uninfected children: haptoglobin (HPT) concentrations were significantly lower (Fig. 1d) and haem, haemopexin (HPX), and haem oxygenase-1 (HO-1) concentrations were significantly higher (Fig. 1e–g) among infected children. Haemolysis, as defined by raised plasma haem and HPT concentrations, was persistent between days 1 and 35 among infected children.

**Table 1:** *Plasmodium falciparum* densities and haemoglobin genotypes of subclinically infected cohort

ID	Age	Gender	Hb Genotype	*Plasmodium falciparum* (parasites/uL, qPCR)	
				Day 0	Day 35
1	6	F	HbAA	5445	1403
2	7	M	HbAA	583	1180
3	8	F	HbAA	492	1107
4	8	M	HbAA	993	265
5	6	M	HbAS	124	18967
6	8	F	HbAA	236	307
7	9	F	HbAA	2084	145
8	9	F	HbAA	175	386
9	9	M	HbAA	2118	2275
10	9	M	HbAC	2173	1268
11	9	F	HbAC	1012	815
12	6	M	HbAA	3	1
13	9	F	HbSC	37	16
14	8	M	HbAA	396	74
15	8	M	HbAA	237	340
16	5	F	HbAA	81	Resolved
17	9	M	HbAA	156	356
18	7	M	HbAC	185	2
19	9	M	HbAA	296	222
20	8	M	HbAA	1600	1315
21	9	M	ND	1871	1641
22	9	M	HbAC	160	89
23	8	F	ND	78	370
Median	8			296	363

*ND* not determined

### Haemoglobin genotypes and haemolysis parameters in children with subclinical *Plasmodium falciparum* infections

Genetic structural variants of haemoglobin can result in haemolytic anaemia, which may in themselves lead to raised plasma heme and elevated HO-1. Therefore, haemoglobin A, C and S carriage within the infected cohort was determined (Table 1). Fifteen children had normal haemoglobin genotype (HbAA) and four children were haemoglobin C carriers (HbAC). While carriage of homozygous HbC can be protective against severe falciparum infection [31, 32], HbC trait does not affect steady state haemoglobin concentration or red cell counts [33]. In agreement with this, levels of haemoglobin, heme, and HO-1 were not statistically different between infected children with hemoglobin HbAA and HbAC genotypes (Table 2). One child carried sickle-cell trait (HbAS) but their Hb, heme and HO-1 values were within the range for the HBAA children at day 0 and day 35. However, the one child who was heterozygous for sickle-cell and hemoglobin C (HbSC) had an Hb concentration that was below the range for HbAA children, and an HO-1 concentration that was above the range for HbAA children, on day 0 but not on day 35 (Table 2) suggesting that this genotype may be associated with exacerbated haemolysis.

**Table 2:** Analysis of anaemia and haemolysis in subclinically *Plasmodium falciparum* infected children grouped by haemoglobin genotype

Genotype	HbAA (n = 15)	HbAC (n = 4)	P-value (HbAA v. HbAC)	HbAS (n = 1)	HbSC (n = 1)
	Median (range)	Median (range)		Value	Value
Day 0					
Patients (n)	15	4		1	1
Hb (g/dL)	11.9 (9.8–14.3)	11.4 (10.7–13.8)	0.90	11.2	9.3
Haem (uM)	23.1 (13–142.3)	17.5 (11.7–23.1)	0.19	11.2	21.4
HO-1 (ng/mL)	2.4 (0.3–6.8)	3.3 (1.0–4.2)	0.73	0.6	7.6
Day 35					
Patients (n)	14	4		1	1
Hb (g/dL)	11.1 (9.6–14.9)	11.2 (10.3–13.7)	0.98	12.3	8.8
Haem (uM)	16.2 (8.2–38.2)	16.3 (9.3–22.8)	0.86	10.3	25.5
HO-1 (ng/mL)	0.7 (0.4–4.0)	1.2 (0.1–11.5)	0.82	1.2	2.5
All Dates					
Patients (n)	29	8			
Hb (g/dL)	11.7 (9.6–14.9)	11.4 (10.3–13.8)	0.93	ND	ND
Haem (uM)	17.1 (8.2–142.3)	17.5 (9.3–23.1)	0.44	ND	ND
HO-1 (ng/mL)	1.3 (0.3–6.8)	2.4 (0.1–11.5)	0.57	ND	ND

ND, medians not determined for HbAS (sickle cell carrier) and HbSC (heterozygous sickle-C carrier) as only one patient carried each of these genotypes

P-values for comparisons between HbAA and HbAC (haemoglobin C carriers) determined by Mann–Whitney test

### Low density parasitaemia is not associated with iron deficiency

**Fig. 2:**
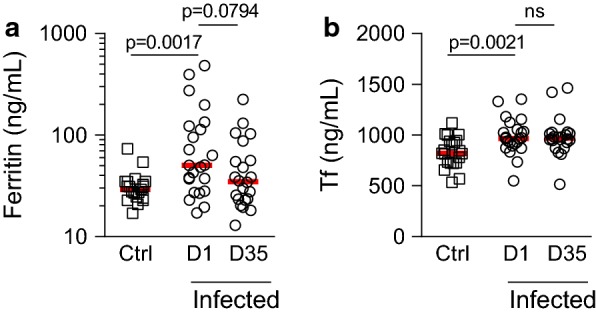
Iron indices during subclinical malaria. Plasma concentrations of (**a**) ferritin or (**b**) transferrin (Tf) determined by ELISA. Dot plots show individual patient parameters. Red lines represent medians. P-values for comparisons between uninfected controls (Ctrl) and infected children at enrollment were determined by Mann–Whitney test. P-values for comparisons between infected children at days 1 and 35 were determined by Wilcoxon matched-pair test. ‘ns’ indicates P > 0.05

The findings of persistent haemolysis in infected children in the absence of anaemia suggests that infected children were able to respond to haemolysis by increasing haemopoesis. This suggests that the children are iron sufficient (defined as ferritin concentration > 30 ng/mL [34]). Indeed, plasma concentrations of both ferritin (indicating iron stores) and transferrin (a high-affinity iron-binding plasma protein which sequesters free iron preventing its uptake by pathogens) were slightly, but significantly, higher among infected children than among controls (Fig. 2).

### Subclinical malaria is associated with raised plasma IL-10 concentrations and IFN-γ is associated with haemolysis

**Fig. 3:**
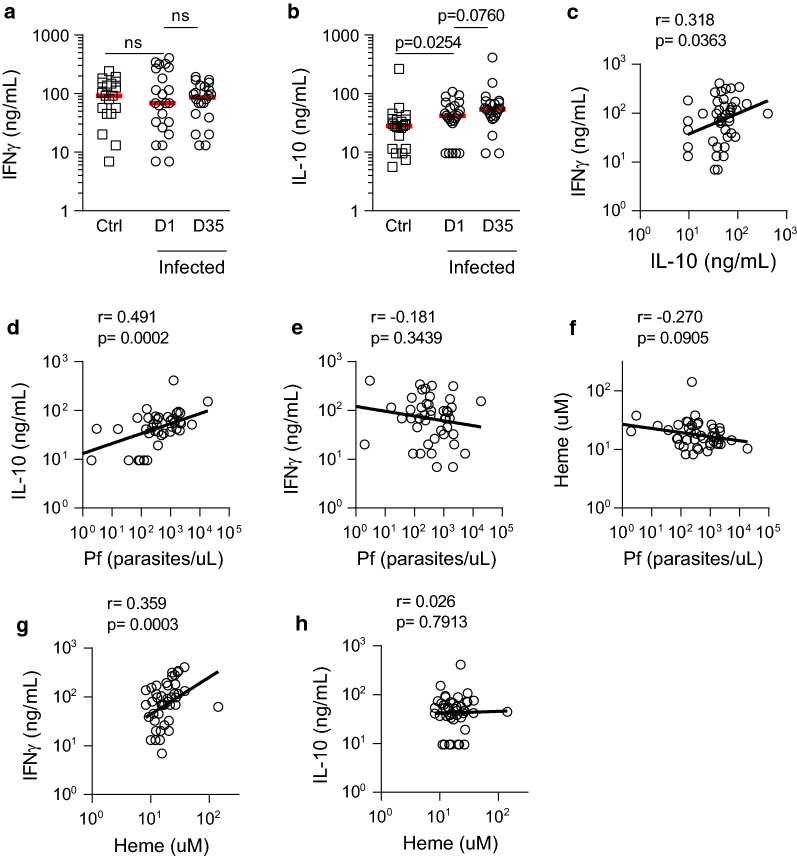
Elevated plasma IL-10 during subclinical malaria. Plasma concentrations of (**a**) interferon gamma (γ) or (**b**) interleukin (IL-) 10 determined by cytokine bead array. Dot plots show individual patient parameters. Red lines represent medians. P-values for comparisons between uninfected and infected children at enrollment were determined by Mann–Whitney test. P-values for comparisons between infected children at days 1 and 35 were determined by Wilcoxon matched-pair test. ‘ns’ indicates P > 0.05. **c**–**h** For infected children, correlations were calculated using log-transformed data (combined D1 and D35). **c** Correlation between IFN-γ and IL-10. Further, parasite density (determined by qPCR) was correlated with (**d**) IL-10, (**e**) IFN-γ and (**f**) haem. Finally, correlations were determined between haem and both (**g**) IFN-γ, and (**h**) IL-10. Log-transformed data shown with linear regression line, Pearson’s correlation coefficient and *p* value; n = 45

Acute malaria infection induces a robust inflammatory response (typically marked by high concentrations of cytokines such as IFN-γ and TNFα) and is accompanied by a compensatory (homeostatic) increase in circulating interleukin (IL)-10) [35–37]. To determine whether low density, subclinical infections induced a similar inflammatory/anti-inflammatory state, plasma IFN-γ and IL-10 concentrations were determined by multiplex bead array. Plasma IFN-γ concentrations did not differ significantly between infected and uninfected children (Fig. 3a) however plasma IL-10 concentrations were modestly but significantly higher in infected children and these raised IL-10 concentrations were sustained during the 35 days of follow up (Fig. 3b).

To understand the possible causal relationships between parasite infection, haemolysis (haem), inflammation (IFN-γ) and immunoregulation (IL-10), associations between these different factors in infected children were calculated, combining observations from days 1 to 35 if qPCR positive (n = 45). Plasma IFN-γ and IL-10 concentrations were positively correlated (r = 0.318, p = 0.04) (Fig. 3c). IL-10 concentration was statistically significantly correlated with qPCR parasite density (r = 0.491, p = 0.0002) (Fig. 3d) while concentrations of IFN-γ (r = − 0.181, p = 0.34) (Fig. 3e) and haem (r = − 0.270, p = 0.09) (Fig. 3f) were not. While IFN-γ concentration was not correlated with parasite density (Fig. 3e), it was positively correlated with haem concentration (r = 0.359, p = 0.0003) (Fig. 3g). In addition, while IL-10 concentration was correlated with parasite density (Fig. 3d), it was not correlated with haem concentration (r = 0.026, p = 0.79) (Fig. 3h).

### HO-1 induction is associated with haemolysis but not parasite density, haemoglobin concentration or IL-10 concentration

**Fig. 4:**
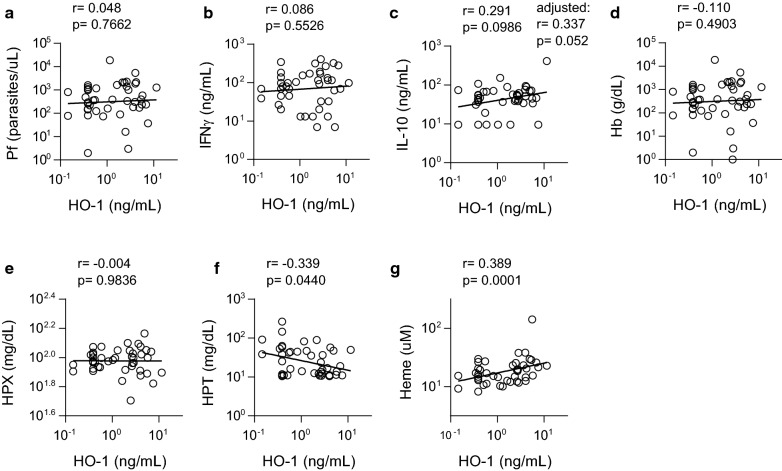
HO-1 induction correlates with markers of haemolysis, but not parasitaemia or anaemia. For infected children, correlations were calculated on log-transformed data (combined D1 and D35). Correlations between haem oxygenase-1 (HO-1) and **a** parasite density, **b** interferon gamma (IFN-γ), **c** interleukin(IL-) 10, **d** haemoglobin (Hb), **e** haemopexin (HPX), **f** haptoglobin (HPT), and **g** haem. Log-transformed data shown with linear regression line, Pearson’s correlation coefficient and p-value; n = 45. Partial correlation coefficient and p-value for the association between IL-10 and HO-1 adjusted for IFN-γ and haem shown in **c**

*Plasmodium falciparum* infection was associated with both haemolysis (Fig. 1) and elevated plasma IL-10 (Fig. 3). HO-1 is directly induced by both free haem (which is released during erythrocyte destruction) and IL-10 [38–41]; induction of HO-1 to reduce haem-induced tissue damage is a major mechanism of IL-10-mediated tissue protection [42]. Accordingly, in infected children, HO-1 concentration was negatively correlated with HPT (r = − 0.339, p = 0.04) and positively correlated with haem (r = 0.389, p = 0.0001) concentrations (Fig. 4f, g). However, there was no significant correlation between parasite density and HO-1 (r = 0.048, p = 0.77) (Fig. 4a). Furthermore, HO-1 concentration was not significantly associated with IFN-γ (r = 0.086, p = 0.55), IL-10 (r = 0.291, p = 0.10), haemoglobin (r = -− 0.110, p = 0.49) or haemopexin (r = − 0.004, p = 0.98) concentrations (Fig. 4b–e).

**Fig. 5:**
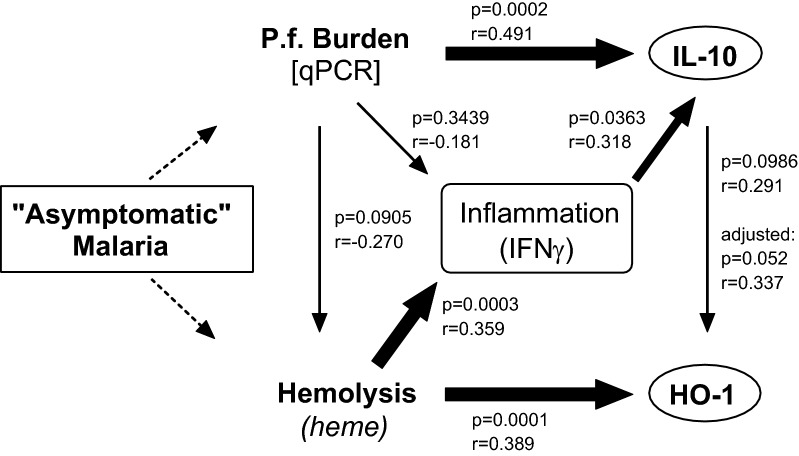
Proposed model of the relation between haemolysis, IFN-γ, IL-10, HO-1 and parasitaemia in subclinically infected children. Approximately half the children with subclinical malaria infection had anaemia, but neither the Hb concentration nor the proportion of those with anaemia differed from uninfected children (Fig. 1). However, those who were PCR positive for *Plasmodium falciparum* had evidence of haemolysis (haem and haemopexin, HPX). Further, while the level of circulating parasites correlated with IL-10 concentration, it did not correlate with free haem, IFN-γ or haem oxygenase-1 (HO-1) induction. Rather, HO-1 levels were positively correlated with circulating free haem and, to a lesser extent, with IL-10 concentrations. Line thickness represents significance level; 6pt = p < 0.001, 3pt = p > 0.001 to p = 0.05, 1/2pt = p > 0.05

These data indicate that parasite density is likely the major driver of IL-10 production while haemolysis is the major driver of HO-1 (proposed model, Fig. 5). After adjusting for haem and IFN-γ concentrations (potential confounders of the association between HO-1 and IL-10, Fig. 5), the correlation between HO-1 and IL-10 became stronger and was of borderline statistical significance (adjusted, r = 0.337, p = 0.05), suggesting that IL-10 may indeed contribute to HO-1 induction, independently of haem. Nevertheless, haem concentration is the strongest predictor of HO-1 concentration in children with subclinical *P. falciparum* infections (r = 0.389, p = 0.0001, Fig. 4g).

## Discussion

The purpose of this study was to begin to test the hypothesis that subclinical *P. falciparum* malaria infections are associated with persistent haemolysis and that this is sufficient to lead to sustained induction of HO-1. The rationale for the study is that-given the documented causal association between HO-1, neutrophil dysfunction and increased susceptibility to non-typhoidal Salmonella bacteraemia [24, 25] -evidence of sustained HO-1 induction in subclinically infected children might explain the epidemiological association between the prevalence of malaria and the incidence of invasive bacterial disease [19, 43, 44]. This would, in turn, justify further studies to understand the impact of subclinical infection on neutrophil function, to describe the nature of any observed neutrophil defects and the potential for treatment of subclinical malaria infections to restore neutrophil function and thereby reduce the incidence of bacteraemia.

In support of this hypothesis, this preliminary study indicated that children with persistent, low density parasite infections show clear evidence of haemolysis (evidenced by raised plasma haem and depleted HPT) which is sustained for at least 35 days as infections persist. Moreover, haemolysis is accompanied by, and highly correlated with, sustained induction of HO-1. This observation highlights the need for larger and more extensive studies of neutrophil function in subclinically infected children.

Clinically, haemolytic anaemia is defined by low haemoglobin concentration accompanied by low HPT and low haemopexin (HPX) [45]. HPT binds haemoglobin and targets it for phagocytic uptake (and degradation) via the CD163 receptor on macrophages. The low HPT concentrations in infected children suggest that haemolysis has been ongoing for sufficient time to deplete available stores of HPT. Conversely, HPX binds cell-free haem and the haem-HPX complex binds to macrophage CD91, again triggering phagocytic uptake and degradation of haem, thereby protecting the body from haem-mediated tissue damage [46]. However, despite evidence of haemolysis, HPX concentrations were in fact higher in subclinically infected children than in uninfected children. This suggests that the levels of haemolysis in these children are sufficient to deplete HPT and induce HO-1 but not sufficient to deplete available stores of HPX. Indeed, these data suggest that modest levels of haemolysis may in fact stimulate the synthesis of HPX. When taken together with the observation that haemoglobin concentrations were not significantly different from those in uninfected children, and that ferritin and transferrin concentrations were within the normal range for healthy children [34, 47], these data suggest that haemolysis is well tolerated in children with subclinical malaria infections and that the children are iron sufficient and able to mount a sufficient erythropoietic response to sustain red cell counts and haemoglobin concentrations. The lack of correlation between parasite density and the extent of haemolysis may be explained by the observation that much of the anaemia associated with malaria is due to lysis or phagocytosis of uninfected erythrocytes as well as parasitized cells [48].

While haemolysis and liberation of free haem induces HO-1, and these data show a clear correlation between plasma concentrations of haem and HO-1, it is important to consider the possibility that IL-10 may also play a role. Malaria-induced inflammation leads to a compensatory secretion of the anti-inflammatory cytokine IL-10 [35] and IL-10 can directly induce HO-1 [38]. In this study of subclinical malaria infections, plasma inflammatory cytokine concentrations did not differ between infected and uninfected children. IL-10 concentrations were, nevertheless, raised in the infected children and correlated with parasite density, confirming many previous studies of circulating cytokine responses to malaria infection [49, 50]. While there was no significant correlation between IL-10 and HO-1 concentrations in univariate analyses, a multivariate model suggested that IL-10 may contribute to HO-1 induction alongside haem. Further work is needed to understand the relative contribution of these two factors to HO-1 induction during low-density infections. One limitation of the current study is that the duration of infections prior to the first qPCR positive observation was unknown and the study was terminated on day 35; it is not known, therefore, how long these children had been infected or how long they had been experiencing haemolysis. More detailed studies are required to ascertain the detailed natural history of subclinical *P. falciparum* infections.

While these preliminary data suggest that subclinical malaria infections induce persistent haemolysis and sustained production of HO-1, the analyses and interpretations are limited by the relatively small sample size and the lack of data on neutrophil function. Moreover, absolute concentrations of HO-1 were lower than previously reported for children recovering from acute febrile malaria [25], raising the possibility that HO-1 levels may not be sufficient to cause neutrophil defects. Further, parasite densities varied by 3 orders of magnitude even among this small, subclinically-infected group. However, this is the first published report of raised plasma haemoxygenase-1 (HO-1) during persistent, subclinical malaria infections and, when taken together with raised plasma IL-10 concentrations, suggests that further studies would be justified to determine the impact of HO-1 and IL-10 on neutrophil function and susceptibility to bacterial infection.

## Conclusions

In this pilot study, children with subclinical malaria infections showed evidence of persistent haemolysis; with raised plasma haem, IL-10 and HO-1. This study supports the growing evidence that these low density malaria infections are not strictly “asymptomatic”. Further work is needed to clarify the impact of these observations on immune status and resilience to co-infection.

## Abbreviations

Hb: haemoglobin
HPT: haptoglobin
HPX: haemopexin
HO-1: haem oxygenase-1
IL-10: interleukin-10
